# Identification of pathogenic fungi causing ocular infections using full rRNA operon sequencing with Oxford Nanopore Technologies

**DOI:** 10.7717/peerj.20997

**Published:** 2026-03-20

**Authors:** Thitima Suwannasaeng, Pratsanee Hiengrach, Waewta Kuwatjanakul, Kittipan Samerpitak, Kiatichai Faksri, Sunchai Payungporn, Pasinee Sangsiwarit, Parama Budmala, Suthida Visedthorn, Pakorn Ruengket, Kanuengnit Srisak, Suwalak Chitcharoen

**Affiliations:** 1 Department of Microbiology, Faculty of Medicine, Khon Kaen University, Khon Kaen, Thailand; 2 Clinical Microbiology Unit, Clinical Laboratory Section, Srinagarind Hospital Faculty of Medicine, Khon Kaen University, Khon Kaen, Thailand; 3 Research and Diagnostic Center for Emerging Infectious Diseases, Khon Kaen University, Khon Kaen, Thailand; 4 Center of Excellence in Systems Microbiology, Department of Biochemistry, Faculty of Medicine, Chulalongkorn University, Bangkok, Thailand

**Keywords:** Ribosomal RNA, Oxford Nanopore Technologies (ONT), Ocular infections, Pathogenic fungi, Internal transcribed Spacer (ITS), Bioinfomatics pipeline, Gene barcoding

## Abstract

Although fungal eye infections are a major cause of visual impairment worldwide, standard clinical laboratory methods remain slow, insensitive, and limited in their taxonomic resolution. Sequencing of the full ribosomal RNA (rRNA) operon provides a comprehensive marker for fungal identification. In this study, twenty fungal isolates associated with ocular infections were obtained from Srinagarind Hospital, Thailand, and characterized using four identification approaches. Initial hospital-based routine identification relied on conventional morphological methods and matrix-assisted laser desorption ionization time-of-flight mass spectrometry (MALDI-TOF MS). To enhance resolution and to develop a comprehensive analytical pipeline, we further employed full rRNA operon sequencing using Oxford Nanopore Technologies (ONT), analyzed through three bioinformatic pipelines: EPI2ME/Minimap2, NGSpeciesID with BLASTn, and internal transcribed spacer (ITS)-based phylogenetic analysis coupled with phylogenetic analysis. All isolates yielded complete operon sequences, thus ensuring comprehensive coverage of the target regions. NGSpeciesID produced high-confidence consensus sequences and species-level classifications for nearly all isolates (except one *Candida* specimen). Of these, 15 of the 20 isolates showed exhibited concordance with hospital identifications at the genus level (≥97% identity). This approach successfully resolved closely related *Aspergillus* taxa (*i.e.*, *A. terreus*, *A. luchuensis*, *A. oryzae*), reclassified *Curvularia* isolates as *Bipolaris maydis*, and confirmed species-level assignments for *Fusarium* and *Rhodotorula*. By contrast, the EPI2ME workflow produced more variable classifications, providing species-level assignments for *Aspergillus* and *Rhodotorula* but mixed genus/species profiles for several isolates, including seven isolate assignments unique to this method. ITS-based phylogenetic reconstruction recovered all expected clades, with *Curvularia* isolates clustering within their genus. However, node support varied substantially, highlighting the limited discriminatory power of ITS alone, which constrains taxonomic resolution to the species-complex level rather than consistently achieving the species-level identification of *Aspergillus* isolates. Overall, ONT-based full-operon sequencing demonstrates strong potential for fungal diagnostics, its performance depends on bioinformatic pipelines, database quality, and sequencing errors. Species-level resolution is particularly limited in *Aspergillus*, while incomplete reference datasets hinder the classification of isolates such as *Curvularia*. To improve reliability and clinical application, it will be essential to expand curated full-length rRNA references, integrate complementary loci, and refine analytical strategies.

## Introduction

Fungal eye infections, including keratitis and endophthalmitis, pose a significant and increasing threat to public health globally, leading to substantial ocular morbidity and vision loss, particularly in tropical and developing regions (Kalkanci & Ozdek, 2011; Mehrandish & Mirzaeei, 2021). Invasive fungal diseases are estimated to cause 1.6 million deaths annually worldwide, and up to half of severe fungal infections remain undiagnosed until autopsy (Brown et al., 2021; Denning, 2024). The impact of fungal eye infections is especially profound, with studies indicating that up to 60% of individuals with fungal keratitis may suffer moderate to severe visual impairment or even blindness (Burton et al., 2011; Sharma et al., 2021).

Despite the critical importance of early diagnosis, traditional diagnostic workflows remain slow, insensitive, and often inadequate. Clinical laboratory methods, including direct microscopic examination, cultivation, and histopathology, only yield positive results in approximately half of cases of fungal eye infection cases, with the production of actionable data taking a long time (Sharma et al., 2021; Mendonça et al., 2022). While approaches such as MALDI-TOF MS and serological assays have shorter turnaround times, their diagnostic accuracy is limited by incomplete fungal databases and poor resolution for closely related species, resulting in frequent misidentification (Singhal et al., 2015; Siller-Ruiz et al., 2017). Although molecular methods such as Sanger sequencing combined with conventional PCR offer high sensitivity and specificity, Sanger sequencing requires purified DNA from a single species and thus cannot reliably identify mixed infections, often typically necessitating prior culture to obtain a pure isolate before sequencing. (Li et al., 2018; Mills et al., 2021; Drevinek et al., 2023). Consequently, delayed or inaccurate diagnoses remain a barrier to effective, sight-saving treatment.

At the core of molecular detection and identification of fungi are ribosomal RNA (rRNA) barcodes. The fungal rRNA operon, which spans 5,000–6,000 base pairs, consists of three conserved rRNA genes (*18S*, *5.8S*, and *28S*) and two hypervariable internal transcribed spacer regions (ITS1 and ITS2) (D’Andreano, Cuscó & Francino, 2021). However, the internal transcribed spacer (ITS) region is widely regarded as the universal fungal barcode (Schoch et al., 2012; Xu, 2016). Nevertheless, ITS alone sometimes lacks sufficient resolution to distinguish between closely related species, particularly within genera, such as *Fusarium* and *Aspergillus* (Stielow et al., 2015; Al-Hatmi et al., 2016; Raja et al., 2017; Kauserud, 2023). Furthermore, alternative markers within the rRNA operon can also be leveraged depending on the phylogenetic diversity and classification needs (Tedersoo et al., 2015; Won, Cho & Kim, 2024).

Simultaneous targeting of the ITS, small subunit (SSU), and large subunit (LSU) regions, particularly with third generation long-read sequencing technologies, enable robust and hierarchically informed identification of diverse clinically significant fungi (Ohta et al., 2023). Recent developments in third-generation sequencing technology, particularly the advent of the Oxford Nanopore Technologies (ONT) platform, are poised to further transform fungal diagnostics. The ONT platform enables rapid and real-time detection of pathogens directly from clinical specimens, thus offering the unique advantage of sequencing ultra-long reads (>100 kb) that can encompass multiple genetic marker regions in a single sequencing run (Jain et al., 2018; Jun et al., 2021; Wang et al., 2021; Chen & Xu, 2023; Ohta et al., 2023). This advancement has the potential to dramatically reduce the time from sample collection to actionable results while improving diagnostic accuracy in clinical settings. Nonetheless, realizing the full diagnostic potential of ONT-based, long-read sequencing requires robust and specialized bioinformatic pipelines. Thus, comprehensive data analysis pipelines are essential to convert complex sequencing outputs into accurate, reproducible, and clinically actionable results, especially in cases where diagnostic speed and reliability are crucial (Langsiri et al., 2023; Hakimzadeh et al., 2024; Bosilj et al., 2024).

Furthermore, effective bioinformatic analysis requires tools that can accommodate the structural complexity inherent to long-read sequencing data and enabling accurate fungal taxonomic identification (Lu et al., 2023). The EPI2ME open-analysis platform is an integrated workflow for analyzing ONT-based sequencing data, enabling per-read taxonomic classification and real-time data interpretation for long-read sequencing datasets (D’Andreano, Cuscó & Francino, 2021). In addition, Minimap2 is a widely used sequence alignment program for long-read sequence data due to its computational efficiency and accuracy in mapping long, error-prone reads against large reference databases, making it well-suited for full-length rRNA operon analyses (Li, 2018). NGSpeciesID is a clustering and consensus-forming tool specifically designed for long-read amplicon sequencing data, employing read clustering and consensus sequence generation to mitigate sequencing errors and enhance species-level resolution, including the detection of mixed-species samples (Sahlin, Lim & Prost, 2021).

Therefore, this study aimed to develop and validate an ONT-based long-read complete rRNA operon sequencing workflow for the identification of pathogenic fungi commonly associated with eye infections. We hypothesized that ONT-based full rRNA operon sequencing would enable rapid and reliable species-level identification, with performance being strongly dependent on the bioinformatics pipeline and reference database used. The workflow included DNA extraction from clinical isolated colonies, polymerase chain reaction (PCR) amplification, ONT-based sequencing, and the comparative evaluation of bioinformatics pipelines. The expected outcomes were a validated, reproducible sequencing and analysis workflow with improved identification accuracy and the identification of critical analytical factors affecting ONT-based fungal diagnostics. Our findings highlight the critical influence of analysis tools and reference databases on identification accuracy.

## Materials & Methods

### Sample collection

Twenty clinical fungal isolates were obtained as part of routine diagnostics from patients at Srinagarind Hospital, Khon Kaen, Thailand. All isolates were identified by routine fungal identification approaches in the hospital. Yeast species, including five *Candida* spp., and four *Rhodotorula* spp., were determined using MALDI-TOF MS (Bruker MALDI Biotyper System; Bruker, Billerica, MA, USA), and filamentous fungi species, including five *Aspergillus* spp., five *Curvularia* spp., and a *Fusarium* spp., were identified based on microscopic morphology. The isolates were cultured on Sabouraud dextrose agar (Oxoid, Basingstoke, Hampshire, UK) and incubated at 37 °C for 2–5 days. All isolates were stored at −20 °C until DNA extraction.

### Fungal DNA extraction

DNA was extracted using the DNeasy UltraClean Microbial Kit (12224-50; QIAGEN, Hilden, Germany) according to the manufacturer’s protocol with minor modifications. Briefly, fungal pellets underwent three rounds of disruption by vortexing at 4,500 rpm with 0.1 mm beads for 3 min, followed by incubation on ice for 3 min. This mechanical disruption was combined with lysis buffers to chemically degrade cellular components. The incubation time with the lysis buffer was extended to 30 min at 70 °C to enhance cell lysis.

### Full rRNA operon amplicon sequencing

PCR amplification of the full-length fungal rRNA operon was performed using primer pair SSU515Fngs-F (5′-GCCAGCAACCGCGGTAA-3′; Tedersoo et al., 2015) and LR12 (5′-ICGACTTAGAGGCGTTCAG-3′ Vigalys, 2018). The 25 µL PCR mixture contained 12.5 µL of KOD One PCR Master Mix (DM015-R500; TOYOBO, Osaka, Japan), 0.75 µL each of the 10 pmol/µL forward and reverse primers, 10 µL of nuclease-free water (Invitrogen, Carlsbad, CA, USA), and one µL of genomic DNA. Thermal cycling was conducted on a T100 Thermal Cycler (Bio-Rad, Hercules, CA, USA) with initial denaturation at 98 °C for 3 min, followed by 35 cycles of 98 °C for 10 sec, 63.8 °C for 5 s (annealing), and 68 °C for 5 sec, ending with a final extension at 68 °C for 5 min. The amplicons (∼6,000 bp) were verified on a 1.5% agarose gel and purified. Next, barcode adapters were added using the PCR Barcoding Expansion Kit (EXP-PBC096; ONT, Oxford, UK), and the products were size-selected using AMPure XP beads (SK2142024; Beckman Coulter, Brea, CA, USA) and quantified using the Qubit dsDNA HS Assay Kit (Invitrogen, Thermo Fisher Scientific, Carlsbad, CA, USA). Then, sequencing libraries were prepared using the Ligation Sequencing Kit (SQK-LSK114; ONT, Oxford, UK). Finally, the library was loaded onto FLO-MIN114 (R10.4.1) flow cells and sequencing using the MinION Mk1B platform, controlled by the MinKNOW software (ONT, Oxford, UK). Basecalling and demultiplexing were performed using the embedded Guppy toolkit (v.6.5.7) and only reads with a minimum quality score of 10 were retained.

### Bioinformatic analyses

Following sequencing, raw reads were analyzed using pipeline (i), which employed the EPI2ME Desktop Application (v.5.2.5) metagenomic workflow (ONT, Oxford, UK) with Minimap2 selected as the classification method against the NCBI 16S_18S_28S_ITS database. Taxonomic filtering was performed by specifying the fungal taxonomic (TaxID: 4751) and applying a minimum percent identity threshold of 97%.

In parallel, the same set of reads was analyzed using pipeline (ii). Sequencing reads underwent length-based filtering with NanoFilt v.2.8.0 (-l 4500 –maxlength 7500), retaining sequences between 4,500 and 7,500 bases in length for downstream analyses (De Coster et al., 2018). Next, the quality-filtered reads were assessed using FastQC v.0.11.9 (Andrews, 2010), which generated per-sample quality metrics. To facilitate efficient evaluation and ensure consistency across multiple datasets, all FastQC reports were aggregated using MultiQC v.1.30 (Ewels et al., 2016). In addition, NanoStat v.1.6.0 was used to summarize overall quality statistics (De Coster et al., 2018), providing a comprehensive overview of sequencing quality.

Subsequently, filtered reads underwent consensus sequence construction using the NGSpeciesID pipeline v.0.3.1 (Sahlin, Lim & Prost, 2021), which clusters and polishes long-read amplicons to produce accurate consensus sequences representing operational taxonomic units (OTUs). The tool was run using the command NGSpeciesID –ont –consensus –medaka –t 24 –fastq, enabling ONT-specific processing, consensus building, and polishing with Medaka v.2.0.1 (Oxford Nanopore Technologies, 2018). Both the alignment results and consensus sequences were compared to the NCBI Fungal Reference Sequence (RefSeq) database (https://ftp.ncbi.nlm.nih.gov/genomes/refseq/fungi/) using BLAST v. 2.12.0+ (Altschul et al., 1990) to achieve species-level taxonomic identification. The top BLASTn hits and associated alignment statistics were used to identify fungal species present in the isolates with high confidence (>90% sequence identity). The complete bioinformatic analysis workflow is publicly available at https://github.com/Thitimasws/Fungi/tree/main.

### Phylogenetic analysis

Phylogenetic analyses were performed using pipeline (iii) to assess evolutionary relationships among fungal isolates based on ITS region sequences. Consensus sequences generated by the NGSpeciesID pipeline were aligned using the ClustalW algorithm (Thompson, Higgins & Gibson, 1994) as implemented in BioEdit (Hall, 1999). Phylogenetic trees were inferred using the maximum likelihood (ML) method (Felsenstein, 1981) in MEGA (v.12; Kumar et al., 2024), applying the best-fitting nucleotide substitution model. The robustness of the inferred topologies was evaluated by bootstrap analysis with 1,000 replicates. The resulting tree resolved distinct clades corresponding to the identified fungal species, confirming the concordance between sequence-based identifications and phylogenetic relationships.

### Ethics statement

The research was conducted after obtaining the approval of Khon Kaen University Ethics Committee for Human Research based on the Declaration of Helsinki and the ICH Good Clinical Practice Guidelines, Khon Kaen University on HE671585.

### Data Availability

All raw sequencing data have been deposited in the NCBI Sequence Read Archive (SRA) under BioProject accession number PRJNA1306083.

## Results

### Fungal species identification based on standard clinical laboratory methods

The MALDI-TOF MS mass spectrometry and microscopic morphological assessment of 20 clinical fungal isolates are shown in Table S1. MALDI-TOF MS enabled species-level identification of isolated yeast, detecting *Candida albicans* in five isolates (C01-C05) and *Rhodotorula mucilaginosa* in four isolates (R01–R04). Lactophenol cotton blue staining was used for filamentous fungi, and their microscopic morphology was examined. This approach provided genus-level resolution for *Aspergillus* spp. in five isolates (A01–A05), *Fusarium* spp. in one isolate (F05), and *Curvularia* spp. in five isolates (Cu01–Cu05).

### Sequencing output and quality filtering of clinical fungal isolates

The 20 clinical fungal isolates yielded successful full-length rRNA operon sequences using the ONT MinION platform. Across isolates, raw read counts ranged from 14,930 to 31,705 reads, with average read lengths of 2,244 to 3,683 bp. After quality and length filtering, the read counts ranged from 6,537 to 16,299 reads, and the average read lengths changed to 4,784 to 5,021 bp (Table 1 and Table S2).

**Table 1:** Sequencing metrics for full-length rRNA operon reads from ONT across clinical samples.

		**Raw read**				**Filtered read**			
**Group**	**n Samples**	**Total reads (** **Mean ± SD)**	**Mean** **read length** **(bp** **± SD** **)**	**Median read length** **(bp** **± SD** **)**	**Mean quality (** **Phred score** **± SD)**	**Total reads (** **Mean ± SD)**	**Mean** **read length (bp** **± SD** **)**	**Median** ** read length (bp** **± SD** **)**	**Mean quality** **(Phred score ± SD)**
C	5	14,930.4 ± 2,357	2,616.2 ± 339	3,428.2 ± 1821	15.3 ± 0.1	6,536.6 ± 1,272	5,020.5 ± 167	5,049.2 ± 168	15.0 ± 0.0
R	4	26,104 ± 8,537	2,244.1 ± 431	1,486.0 ± 1,589	14.8 ± 0.1	9,120.8 ± 2,856	4,854.9 ± 1.2	4,857.2 ± 1.5	15.1 ± 0.1
A	5	31,704.8 ± 17,377	2,379.0 ± 696	1,607.0 ± 1,805	14.7 ± 0.1	10,387.4 ± 1,392	4,829.8 ± 12.8	4,831 ± 13.1	14.8 ± 0.0
F	1	26,142.0	3,683.2	4,771.0	14.7	16,299.0	4,783.9	4,786	14.8
Cu	5	16,052.2 ± 4,949	3,021.9 ± 897	3,177.0 ± 1,872	14.8 ± 0.1	8,714.6 ± 2,286	4,814.3 ± 18.7	4,815.8 ± 18.4	15.0 ± 0.0

**Notes.**

C *Candida* spp. R *Rhodotorula* spp. A *Aspergillus* spp. F *Fusarium* spp. Cu *Curvularia* spp. bp base pairs SD standard deviation

### Fungal species identification by full-length rRNA operon sequencing

**Figure 1:**
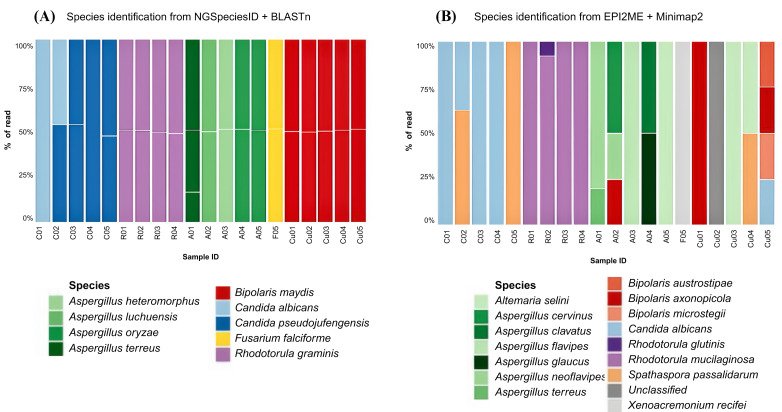
Identification of fungal species from clinical isolates using two bioinformatics pipelines. (A) Proportion of reads assigned to fungal species in each sample using NGSpeciesID combined with BLASTn. (B) Proportion of reads assigned to fungal species in each sample using EPI2ME combined with Minimap2. Different colors represent distinct species-level assignments. Clinical isolate sample IDs are shown on the *x*-axis, and the percentage of reads mapped to each species is indicated on the *y*-axis.

Taxonomic classification using the NGSpeciesID + BLASTn pipeline (Fig. 1A) achieved species-level resolution for all isolates, with 100% read support for every isolate except for a single mixed *Candida* isolate (C02), which showed an equal read distribution (46.7:53.3) between *C. albicans* and *C. pseudojiufengensis*. This workflow reliably identified *R. graminis*, *Aspergillus* spp., and *F. falciforme*, and reclassified all *Curvularia* isolates as *Bipolaris maydis*. In contrast, the EPI2ME + Minimap2 pipeline (Fig. 1B) produced more heterogeneous and occasionally discordant taxonomic assignments, including mixed-species profiles, alternative genus/species identifications (*e.g.*, *Spathaspora passalidarum*, *Alternaria selini*, and *Xenoacremonium recifei*), and instances of unclassified reads. The discrepancies were most evident in the *Aspergillus*, *Fusarium*, and *Curvularia* genera.

### Fungi identification based on the ITS region using phylogenetic tree analysis

**Figure 2:**
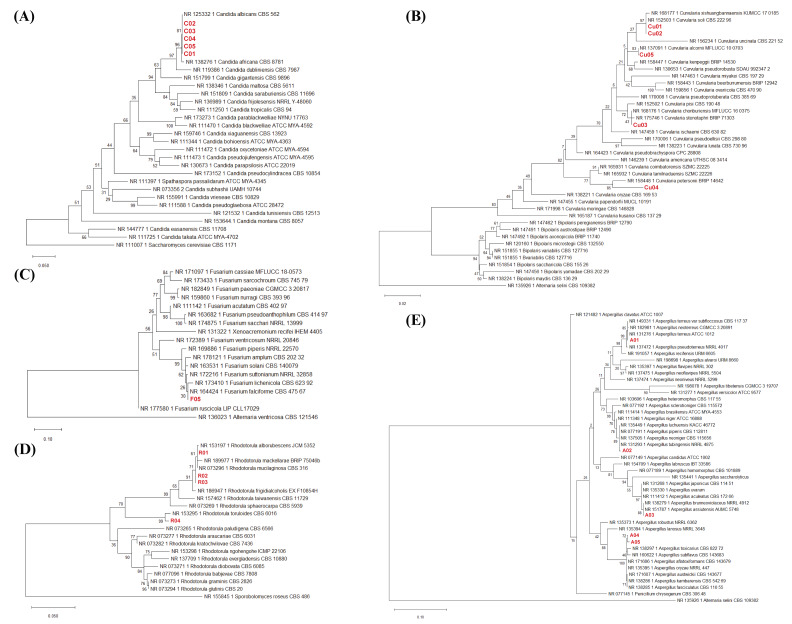
The maximum-likelihood (ML) phylogenetic trees of all isolates based on the ITS region obtained from clinical isolates and reference strains. ML phylogenetic trees for (A) *Candida* spp., (B) *Curvularia* spp., (C) *Fusarium* spp., (D) *Rhodotorula* spp., and (E) *Aspergillus* spp. Clinical isolates obtained in this study are shown in red and compared with reference strains retrieved from the NCBI database. Bootstrap values from 1,000 replications are indicated at the nodes.

Phylogenetic trees were constructed to evaluate ITS-based taxonomic classification for each of the five genera. The ITS-based maximum-likelihood phylogenetic trees, with associated bootstrap support values, are shown in Figs. 2A–2E. The *Candida* isolates (C01–C05) consistently grouped within the *C. albicans* clade, with strong bootstrap support (81%) and no observed sequence divergence (distance = 0.000) across 696 aligned positions (Fig. 2A, Data S1). The *Curvularia* isolates (Cu01–Cu05) resolved into four distinct lineages (702 aligned positions; Fig. 2B, Data S1). Isolates Cu01 and Cu02 were clustered with *C. soli* (97%, 0.000), isolate Cu03 was clustered with *C. chonburiensis* (43%, 0.000), isolate Cu04 was aligned with *C. petersonii* (85%, 0.025), and isolate Cu05 was clustered with *C. alcornii* (83%, 0.000).

The *Fusarium* isolate (F05) was assigned to the *F. solani* species complex, supported by a high bootstrap value (99%). Within this complex, isolate F05 clustered most closely with *F. falciforme*, although with low bootstrap support (30%, Fig. 2C). However, the absence of sequence divergence (distance = 0.000) was only observed between F05 and *F. falciforme* based on 547 aligned positions (Fig. 2C, Data S1).

The *Rhodotorula* isolates (R01–R04) formed two major clusters (598 aligned positions: Fig. 2D, Data S1). Isolate R01 was clustered with *R. mucilaginosa* (61%, 0.000), while isolates R02 and R03 were also resolved as *R. mucilaginosa* but with higher bootstrap support (71%, 0.002). Isolate R04 was clustered distinctly with *R. toruloides* (99%, 0.011).

The *Aspergillus* isolates (A01–A05) were separated into four clades (612 aligned positions; Fig. 2E, Data S1). Isolate A01 was clustered with *A. terreus var. subfloccosus* and *A. terreus* (85%, 0.000), isolate A02 formed a cluster with *A. luchuensis*, *A. piperis*, *A. tubingensis*, and *A. neoniger* (89%, 0.003), isolate A03 was clustered with *A. assisutensis* and *A. brunneoviolaceus* (88%, 0.000), while isolates A04 and A05 were clustered with *A. oryzae* and *A. aflatoxiforman* (72%, 0.003–0.007).

Overall, the ITS-based phylogenetic analyses supported genus-level and, in several cases, species-level affiliations for *Candida*, *Curvularia*, *Fusarium*, and *Rhodotorula* isolates. Species-level assignments were primarily supported by pairwise genetic distance metrics (Data S1) rather than bootstrap support alone. In contrast, *Aspergillus* isolates exhibited multiple species-level affiliations within broader clades, thus highlighting the limited resolving power of ITS-based phylogeny for closely related or cryptic species within this genus.

### Comparison of clinical laboratory with ONT-based taxonomic classification

**Figure 3:**
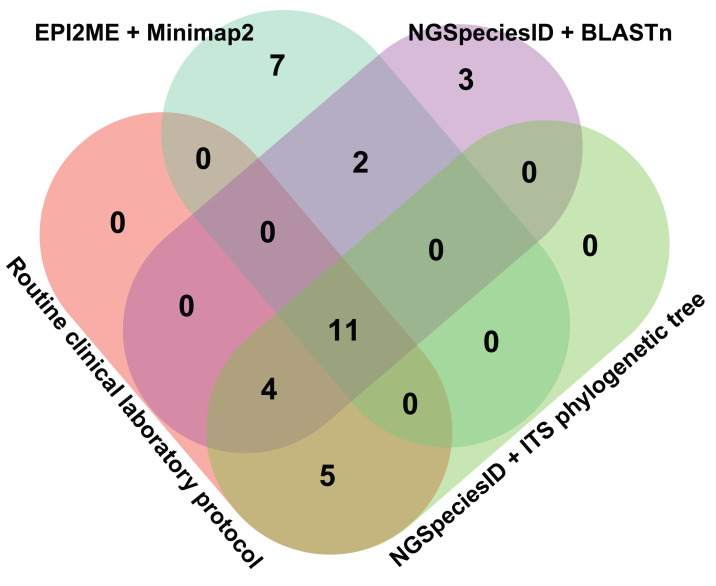
Venn diagram of fungal species identified by different bioinformatics pipelines. The diagram showing the number of fungal species identified uniquely or commonly across four analysis pipelines: EPI2ME + Minimap2, NGSpeciesID + BLASTn, NGSpeciesID + ITS phylogenetic tree, and the routine clinical laboratory protocol. Numbers indicate the count of isolates in which taxa were detected at the genus level for each subset.

Fungal identification was compared between the routine hospital protocol (including microscopic morphology and MALDI-TOF MS) and three ONT-based bioinformatics pipelines: NGSpeciesID with BLASTn, NGSpeciesID with ITS phylogenetic tree analysis, and the EPI2ME metagenomic workflow with Minimap2 (Fig. 3). At the genus level, 11 isolates were consistently identified across all four approaches, representing the genera *Candida*, *Rhodotorula*, and *Aspergillus*. The only three-methods overlap was *Fusarium* (one isolate), identified by the routine hospital protocol, NGSpeciesID + BLASTn, and the ITS phylogenetic tree pipeline. Pairwise overlaps included *Curvularia* (four isolates), identified only by the routine hospital protocol and the ITS phylogenetic tree pipeline, and *Bipolaris* (two isolates) identified only by the NGSpeciesID + BLASTn and EPI2ME + Minimap2 pipeline. Unique detections were restricted to the EPI2ME + Minimap2 pipeline, which identified *Spathaspora*, *Alternaria*, and *Xenoacremonium* (seven isolates altogether). In total, 20 isolates were distributed across the four methods, with the ONT-based pipelines detecting additional genera not captured by the routine hospital protocol.

Comparisons of the routine clinical laboratory protocol with the ONT-based pipelines showed variable concordance across isolates (Table 2). Using the NGSpeciesID + BLASTn pipeline, 15 of the 20 isolates achieved genus-level matches consistent with the routine hospital protocol, with ≥97% sequence identity. Discrepancies were most evident among *Candida* and *Rhodotorula* isolates. While the routine hospital protocol classified all five *Candida* isolates as *C. albicans*, BLASTn frequently assigned them to *C. pseudojiufengensis* (∼93% identity), and *R. mucilaginosa* was often reassigned to *R. graminis* (∼97%). For the *Aspergillus* isolates, BLASTn identified them as species such as *A. terreus*, *A. luchuensis*, and *A. oryzae* with high identity (≥97%), compared to the genus-only identification by the routine hospital protocol. The EPI2ME + Minimap2 pipeline produced more variable outputs, often returning mixed or unrelated taxa (*e.g.*, *Spathaspora passalidarum* for *Candida*, *Xenoacremonium recifei* for *Fusarium*, and *Alternaria selini* for *Curvularia*). The ITS phylogenetic pipeline showed that most *Candida*, *Rhodotorula*, *Aspergillus, Curvularia*, and *Fusarium* isolates clustered within their respective species complexes. Notably, all five *Curvularia* isolates were reclassified as *B. maydis* (98% identity) by BLASTn, whereas the ITS phylogenetic tree consistently retained them within *Curvularia* clade, with bootstrap support ranging from 30% to 99%.

**Table 2:** Comparative fungal identification results from standard clinical laboratory methods, EPI2ME with Minimap2 metagenomic workflow, NGSpeciesID with BLASTn analysis, and NGSpeciesID with ITS-based phylogenetic tree pipeline.

**Sample ID**	**Standard clinical laboratory methods**	**Full rRNA with EPI2ME + Minimap2**	**Full rRNA with NGSpeciesID + BLASTn (% identity)**	**ITS with NGSpeciesID + ITS phylogenetic trees (%Bootstrap value, distance value)**
C01	*C. albicans*	*C. albicans*	*C. albicans* (99.96)	*C. albicans* (81, 0.000)
C02	*C. albicans*	*S. passalidarum*	*C. albicans* (99.82)	*C. albicans* (81, 0.000)
C03	*C. albicans*	*C. albicans*	*C. pseudojiufengensis* (93.39)	*C. albicans* (81, 0.000)
C04	*C. albicans*	*C. albicans*	*C. pseudojiufengensis* (93.48)	*C. albicans* (81, 0.000)
C05	*C. albicans*	*S. passalidarum*	*C. pseudojiufengensis* (93.45)	*C. albicans* (81, 0.000)
R01	*R. mucilaginosa*	*R. mucilaginosa*	*R. graminis* (97.13)	*R. mucilaginosa* (61, 0.000)
R02	*R. mucilaginosa*	*R. mucilaginosa*	*R. graminis* (97.15)	*R. mucilaginosa* (71, 0.002)
R03	*R. mucilaginosa*	*R. mucilaginosa*	*R. graminis* (97.13)	*R. mucilaginosa* (71, 0.002)
R04	*R. mucilaginosa*	*R. mucilaginosa*	*R. graminis* (96.99)	*R. toruloides* (99, 0.011)
A01	*Aspergillus* sp.	*A. neoflavipes*	*A. terreus* (99.51)	*A. terreus var subfloccosus*, *A.terreus* (85, 0.000)
A02	*Aspergillus* sp.	*A. cervinus*	*A. luchuensis* (99.92)	*A. luchuensis, A.piperis A. tubingensis, A. neoniger* (89, 0.003)
A03	*Aspergillus* sp.	*A. flavipes*	*A. heteromorphus* (97.42)	*A. assisutensis, A. brunneoviolaceus* (88, 0.000)
A04	*Aspergillus* sp.	*A. clavatus*	*A. oryzae* (99.90)	*A. oryzae, A. aflatoxiforman* (72, 0.003)
A05	*Aspergillus* sp.	*A. selini*	*A. oryzae* (99.92)	*A. oryzae, A. aflatoxiforman* (72, 0.007)
F05	*Fusarium* sp.	*X. recifei*	*F. falciforme* (99.94)	*F. falciforme* (30, 0.000)
Cu01	*Curvularia* sp.	*B. axonopicola*	*B. maydis* (98.35)	*C. soli* (97, 0.000)
Cu02	*Curvularia* sp.	Unclassified	*B. maydis* (98.24)	*C. soli* (97, 0.000)
Cu03	*Curvularia* sp.	*A. selini*	*B. maydis* (98.31)	*C. chonburiensis* (43, 0.000)
Cu04	*Curvularia* sp.	*A. selini*	*B. maydis* (98.22)	*C. petersonii* (85, 0.025)
Cu05	*Curvularia* sp.	*B. austrostipae*	*B. maydis* (98.17)	*C. alcornii* (83, 0.000)

## Discussion

Fungal infection diagnosis has traditionally targeted a limited set of common pathogens. However, the expanding diversity of fungi linked to human disease highlights the need for rapid, accurate, and broad-spectrum taxonomic profiling methods (Ohta et al., 2023). The genomic region chosen for analysis can depend on the fungal species and the desired level of taxonomic distinction; for example, the SSU and LSU regions are suitable for identifying fungi to at least the family level, while the ITS regions offer higher resolution at lower taxonomic ranks. Using primers to amplify the D1-D2 domains of the LSU region can produce fragments that enable more accurate species-level assignment (Raja et al., 2017; D’Andreano, Cuscó & Francino, 2021). Sanger sequencing of the *18S* ribosomal RNA (rRNA) gene, which is commonly used for higher-level taxonomic identification, requires more than six individual sequencing reactions to span the entire locus (Wu, Xiong & Yu, 2015; Drevinek et al., 2023).

Notably, our study employed an ONT-based full-length rRNA operon sequencing approach, enabling simultaneous coverage of the *18S* (SSU), *28S* (LSU), and ITS regions within a single read, thereby providing a broader and more integrated taxonomic signal than conventional single- or partial-region approaches (Ohta et al., 2023). ONT-based sequencing overcomes the limitations of existing methods by capturing the entire rRNA operon in a single run, reducing errors, minimizing handling, and enabling multi-level taxonomic classification. It captures the *18S* rRNA gene for higher-order relationships and the *28S* rRNA gene and ITS regions for species-level resolution, without conflicts that may arise from separate assays (D’Andreano, Cuscó & Francino, 2021). While Ohta et al. (2023) demonstrated the phylogenetic resolution achievable using nanopore rRNA sequencing, the present study builds upon this by leveraging extended full-length rRNA operon amplicons and systematically comparing multiple bioinformatic pipelines, enabling assessment of how analytical strategy and reference database choice influence species-level identification in a clinical context.

From a practical perspective, the current ONT-based workflow enables species-level identification within approximately 6 h from DNA extraction to bioinformatic analysis. However, the overall diagnostic turnaround time remains constrained by the need for fungal culturing, which typically takes 2–5 days depending on the species, highlighting an important limitation for rapid clinical decision-making. Notably, Oxford Nanopore sequencing has demonstrated potential for direct pathogen detection from clinical specimens without prior culture, which could substantially reduce diagnostic turnaround time and minimize culture-associated bias (D’Andreano, Cuscó & Francino, 2021). Integration of direct-from-specimen sequencing with full-length rRNA operon analysis, therefore, represents a promising future direction for rapid and comprehensive fungal diagnostics. From an economic standpoint, while the initial instrument cost for Sanger sequencing is generally higher than that for ONT platforms, per-run reagent costs are higher for ONT-based sequencing than for Sanger sequencing (Goodwin, McPherson & McCombie, 2016; Van Dijk et al., 2018). Nevertheless, ONT-based sequencing becomes increasingly cost-effective when multiple samples are processed simultaneously or when multi-locus or full-length rRNA operon information is required within a single assay, highlighting its scalability and potential clinical utility in reference and high-throughput diagnostic settings (Deamer, Akeson & Branton, 2016; Jain et al., 2018).

Despite these advantages, the full-length rRNA operon representation in public reference databases remains limited. While ITS datasets are relatively comprehensive for many fungal groups, curated collections of full-length rRNA operon sequences remain scarce (Lu et al., 2023; Ohta et al., 2023). Expanding these resources with high-quality, taxonomically validated reference sequences would enhance classification accuracy by enabling alignment across the entire rRNA operon, without the redundancy and storage demands of whole-genome datasets. Such curated references are particularly valuable in clinical contexts, where precise species-level identification informs targeted treatment strategies (Halwachs et al., 2017; Lücking et al., 2020).

Our study compared four fungal identification strategies; (1) the routine hospital protocol based on microscopic morphology and MALDI-TOF MS, (2) full-length rRNA operon sequencing using NGSpeciesID for clustering and BLASTn-based identification against the NCBI fungal RefSeq database, (3) full-length rRNA operon sequencing with the EPI2ME metagenomic workflow and Minimap2 alignment against the NCBI 16S_18S_28S_ITS database, and (4) ITS phylogenetic tree analysis. The NCBI fungal RefSeq database is a curated repository comprising high-quality, complete or near-complete sequences, including full-length rRNA operons and whole genomes. This curation improves taxonomic classification specificity and reliability, particularly when aligned with long, high-fidelity ONT reads (O’Leary et al., 2016). While the EPI2ME database is broader in scope and sequence volume, it is predominantly composed of short, partial-region sequences, and lacks fungal-specific curation, thereby reducing discriminatory power and increasing the likelihood of ambiguous taxonomic assignments (Pedroso-Roussado et al., 2023).

Importantly, species identification using NGSpeciesID in this study was based on high-depth consensus sequences (>1,000 reads per sample), whereas previous studies indicate that reliable consensus-based identification can be achieved with ≥300 reads per cluster (Ohta et al., 2023). This consensus-based strategy effectively mitigates residual sequencing errors, enhances sequence accuracy, and improves taxonomic reliability, thereby minimizing taxonomic misassignments associated with single-read errors (Sahlin, Lim & Prost, 2021). In contrast, the EPI2ME workflow classifies each read individually without clustering, increasing sensitivity but often generating multiple, inconsistent assignments per sample (Tikhonov, Leach & Wingreen, 2015). Our findings revealed that the EPI2ME-based pipeline produced unsupported identifications, such as *S. passalidarum*, which is consistent with previous reports that per-read classification can overestimate species richness or misassign taxa due to sequencing errors or limited reference data (Langsiri et al., 2023; Shi et al., 2023).

Remaining discrepancies, most evident among *Candida , Curvularia,* and *Aspergillus* isolates, likely stem from the high conservation of the rRNA operon and incomplete reference sequences databases (White et al., 1990; Pryszcz et al., 2014). Additionally, individual-read alignments are susceptible to rare sequencing errors and low-level contamination, which may result in spurious taxonomic assignments, particularly for genera with limited representation in high-quality reference databases. Moreover, ITS-based phylogenetic analysis further highlighted these challenges. Although it resolved major clades and species across the *Candida*, *Curvularia*, *Fusarium*, and *Rhodotorula* genera, species-level placement remained uncertain for *Aspergillus* isolates. These isolates were distributed across multiple clades, illustrating how ITS-based phylogenies can delineate complexes but not reliably distinguish species, especially for *Aspergillus* spp. (Peterson, 2008; Watanabe et al., 2011). These limitations are further exacerbated when relying on conserved loci such as *18S* or *28S* rRNA genes, which provide broad phylogenetic signal but insufficient resolution to distinguish closely related or cryptic species (Stielow et al., 2015; Yarza et al., 2017). To address these challenges, multi-gene barcoding strategies incorporating housekeeping genes such as β-tubulin (BenA), translation elongation factor 1-α (TEF1-α), and calmodulin (CaM), have been increasingly adopted in fungal systematics. These protein-coding genes evolve more rapidly than rRNA markers, providing complementary phylogenetic information that can resolve species boundaries within species complexes such as *Aspergillus* genera (Samson et al., 2014; Stielow et al., 2015; Raja et al., 2017; Kauserud, 2023).

Full-length rRNA classification was particularly effective for *Aspergillus* spp., with NGSpeciesID consistently resolving species such as *A. terreus*, *A. luchuensis*, and *A. oryzae*. This finding aligns with previous reports highlighting the superior discriminatory power of full-length rRNA datasets for this genus (Ohta et al., 2023). Clinically, accurate species-level identification is essential, as closely related species within the same genus may exhibit distinct antifungal susceptibility profiles that influence therapeutic decisions. For instance, *A. terreus* is intrinsically resistant to amphotericin B, unlike *A. fumigatus*, and misidentification can lead to treatment failure (Posch et al., 2018; Lass-Flörl et al., 2021). In our study, several species such as *A. neoterreus*, *A. neoniger*, *A. brunneoviolaceus*, and *A. austwickii* currently lack Clinical and Laboratory Standards Institute (CLSI) breakpoints or epidemiological cutoff values (ECVs) (Lockhart, Ghannoum & Alexander, 2017). The misapplication of ECVs from other species may lead to misclassification of susceptibility and inappropriate antifungal selection, potentially compromising patient outcomes (Espinel-Ingroff et al., 2016; Lockhart, Ghannoum & Alexander, 2017; Dannaoui & Espinel-Ingroff, 2019). These findings underscore the importance of precise species-level identification for future treatment strategies, epidemiological surveillances, and research.

Despite these strengths, our study had some limitations. Firstly, the sample size was relatively small (20 isolates) and was derived from a single tertiary hospital, which may limit the generalizability of our findings to broader clinical contexts. Secondly, although ONT-based sequencing allowed full-length rRNA operon coverage, the accuracy of species-level identification was restricted by the availability and completeness of reference sequences. Indeed, while ITS datasets are relatively well represented, curated full-length *18S* and *28S* rRNA reference sequences remain limited in public repositories. These gaps limit the full potential of rRNA operon-based phylogenetic analysis and highlight the urgent need to expand curated databases with high-quality full-length rRNA operon sequences (Ohta et al., 2023).

Overall, accurate fungal species identification in ocular infections is essential but remains constrained by time-intensive diagnostic workflows and the limitations of available reference databases. ONT-based full-length rRNA operon sequencing represents a promising solution to overcome these challenges. However, continued development of comprehensive databases and optimized analytical pipelines will be essential to maximize their diagnostic utility.

## Conclusions

We analyzed ocular infection–associated fungi from five genera in 20 clinical samples using four identification approaches, demonstrating the utility of ONT-based full-length rRNA operon sequencing for precise species-level identification. This integrated workflow, combining stringent quality control, advanced long-read alignment, high-resolution consensus sequence generation, and robust reference-based identification, provides a reliable framework for fungal species identification using ONT-based sequencing data. Full-length rRNA operon targeting produced high-quality reads, enabling robust classification, although taxa with close phylogenetic relationships (*e.g.*, *Curvularia*) remain challenging to identify. Species-level resolution is vital for *Aspergillus* because of differences in intrinsic antifungal resistance, which directly affect therapy. Discrepancies among the examined analytical pipelines reflected variations in bioinformatics strategies, alignment tools, and database completeness, highlighting the need for expanded, high-quality full-length rRNA reference datasets to improve fungal taxonomy, diagnostics, and patient care.

## Supplemental Information

10.7717/peerj.20997/supp-1 Supplemental Information 1 Fungal species identified by conventional culture-based diagnostics in 20 ocular isolates

10.7717/peerj.20997/supp-2 Supplemental Information 2 Sequencing statistics for full-length rRNA operon reads from ONT across clinical samples

10.7717/peerj.20997/supp-3 Supplemental Information 3 Distance matrix of five fungal genera derived from ITS-based phylogenetic trees of clinical and reference isolates

## Additional Information and Declarations

### Data Availability

The following information was supplied regarding data availability: The raw sequencing data are available in the NCBI Sequence Read Archive (SRA) BioProject: PRJNA1306083.
