# Supplementary material for: Identification of pathogenic fungi causing ocular infections using full rRNA operon sequencing with Oxford Nanopore Technologies
Source: PeerJ. 2026 Mar 20;14:e20997. doi: 10.7717/peerj.20997 (PMC13007636; doi:10.7717/peerj.20997)
Supplement: Supplemental Information 1 [file peerj-14-20997-s001.docx]

**Supplementary Table S1:**

**Fungal species identified by conventional culture-based diagnostics in 20 ocular isolates.**

| **Sample ID** | **Routine hospital approach** | **Result** |
| --- | --- | --- |
| C01 | MALDI-TOF | *Candida albicans* |
| C02 | MALDI-TOF | *Candida albicans* |
| C03 | MALDI-TOF | *Candida albicans* |
| C04 | MALDI-TOF | *Candida albicans* |
| C05 | MALDI-TOF | *Candida albicans* |
| R01 | MALDI-TOF | *Rhodotorula* *mucilaginosa* |
| R02 | MALDI-TOF | *Rhodotorula* *mucilaginosa* |
| R03 | MALDI-TOF | *Rhodotorula* *mucilaginosa* |
| R04 | MALDI-TOF | *Rhodotorula* *mucilaginosa* |
| A01 | Microscopic morphology | *Aspergillus* sp. |
| A02 | Microscopic morphology | *Aspergillus* sp. |
| A03 | Microscopic morphology | *Aspergillus* sp. |
| A04 | Microscopic morphology | *Aspergillus* sp. |
| A05 | Microscopic morphology | *Aspergillus* sp. |
| F05 | Microscopic morphology | *Fusarium* sp. |
| Cu01 | Microscopic morphology | *Curvuralia* sp. |
| Cu02 | Microscopic morphology | *Curvuralia* sp. |
| Cu03 | Microscopic morphology | *Curvuralia* sp. |
| Cu04 | Microscopic morphology | *Curvuralia* sp. |
| Cu05 | Microscopic morphology | *Curvuralia* sp. |
