# Supplementary material for: Identification of pathogenic fungi causing ocular infections using full rRNA operon sequencing with Oxford Nanopore Technologies
Source: PeerJ. 2026 Mar 20;14:e20997. doi: 10.7717/peerj.20997 (PMC13007636; doi:10.7717/peerj.20997)
Supplement: Supplemental Information 2 [file peerj-14-20997-s002.docx]

**Supplementary Table S2:**

**Sequencing statistics for full-length rRNA operon reads from ONT across clinical samples.**

| **Sample** | **Raw read** | | | | | **Filtered read** | | | |
| --- | --- | --- | --- | --- | --- | --- | --- | --- | --- |
|  | Total Reads | Mean Read Length | Median Read Length | Mean Quality | Total Reads | | Mean Read Length | Median Read Length | Mean Quality |
| C01 | 12060 | 2814.8 | 4650 | 15.4 | 6582 | | 4721.8 | 4748 | 15 |
| C02 | 13397 | 2830.1 | 4593 | 15.2 | 6723 | | 5097 | 5126 | 15 |
| C03 | 16426 | 2937.7 | 5005 | 15.2 | 8472 | | 5095.4 | 5125 | 15 |
| C04 | 14776 | 2216.7 | 1261 | 15.2 | 5039 | | 5091.3 | 5122 | 15 |
| C05 | 17993 | 2281.8 | 1632 | 15.3 | 5867 | | 5097 | 5125 | 15 |
| R01 | 29433 | 1991.3 | 545 | 14.6 | 9112 | | 4854.9 | 4858 | 15.1 |
| R02 | 33693 | 2368.3 | 1008 | 14.8 | 13146 | | 4855.9 | 4858 | 15 |
| R03 | 27366 | 1823.3 | 544 | 14.8 | 7441 | | 4855.6 | 4858 | 15.1 |
| R04 | 13924 | 2793.3 | 3847 | 14.8 | 6784 | | 4853.2 | 4855 | 15.1 |
| A01 | 62415 | 1587.7 | 737 | 14.7 | 11107 | | 4843.8 | 4844 | 14.8 |
| A02 | 25233 | 2105.3 | 617 | 14.8 | 8837 | | 4834.7 | 4836 | 14.8 |
| A03 | 27143 | 2248.8 | 726 | 14.6 | 9766 | | 4809 | 4809 | 14.7 |
| A04 | 19808 | 3479.6 | 4817 | 14.7 | 12415 | | 4829.6 | 4832 | 14.8 |
| A05 | 23925 | 2472.1 | 1138 | 14.8 | 9812 | | 4831.9 | 4834 | 14.8 |
| F05 | 26142 | 3683.2 | 4771 | 14.7 | 16299 | | 4783.9 | 4786 | 14.8 |
| Cu01 | 12101 | 2575.4 | 3533 | 15 | 5763 | | 4821.6 | 4823 | 15 |
| Cu02 | 12195 | 3577.8 | 4785 | 14.8 | 8172 | | 4794.8 | 4796 | 15 |
| Cu03 | 19186 | 2174.2 | 316 | 14.8 | 7623 | | 4830.6 | 4832 | 15 |
| Cu04 | 13556 | 4317.9 | 4792 | 14.9 | 11309 | | 4793.8 | 4796 | 15 |
| Cu05 | 23223 | 2464 | 2459 | 14.7 | 10706 | | 4830.8 | 4832 | 15 |
